# Are postnatal traumatic events an underestimated cause of porencephalic lesions in dogs and cats?

**DOI:** 10.3389/fvets.2023.1302399

**Published:** 2023-12-06

**Authors:** Tommaso Davini, Chiara Mattei, Claudia La Rosa, Carlotta Remelli, Swan Specchi, Elena Lionello, Elena Dell’Era, Marco Bernardini

**Affiliations:** ^1^Anicura I Portoni Rossi Veterinary Hospital, Zola Predosa, Bologna, Italy; ^2^Antech Imaging Service, Fountain Valley, CA, United States; ^3^Department of Animal Medicine, Productions and Health, University of Padua, Legnaro, Italy

**Keywords:** Porencephaly, cavitary lesions, cyst-like lesions, congenital brain disease, cerebrospinal fluid (CSF), trauma, magnetic resonance imaging (MRI)

## Abstract

**Introduction:**

Porencephaly is defined as a fluid-filled cavity of variable size in the brain cortex. It is regarded as a congenital condition and is typically considered a developmental or an encephaloclastic defect. Our hypothesis is that postnatal traumatic events in the first few months of life may represent a cause of canine and feline porencephaly that is more common than generally suspected. The aims of this study were to retrospectively investigate porencephaly in a large population of dogs and cats, detect MRI features that might be useful to differentiate postnatal acquired traumatic forms from congenital/perinatal porencephaly, and define the prevalence of seizure activity in porencephalic patients.

**Materials and methods:**

This is a double-center, descriptive, retrospective case series. Databases were searched for cases within a 17-year time span that involve dogs and cats with an MRI-based diagnosis of cerebral cavitary lesions. Animals were included if a complete signalment and an exhaustive MRI of the brain were available. Besides the porencephalic lesions, MRIs of the head were reviewed to detect concomitant musculoskeletal abnormalities.

**Results:**

Thirty-two cases involving nine cats and twenty-three dogs were selected. Of all the cases, 21.9% were aged six years or older at the time of diagnosis. All patients in which the neuroanatomical localization was available showed clinical signs of a prosencephalic disorder. Epileptic seizures were observed in 71.8% of cases. A single porencephalic cavity was found in 78.1% of cases. The most affected cerebral lobe was the parietal lobe (*n* = 20). The defects involved both the grey and white matter in 78.1% of cases. Twenty cases showed concomitant musculoskeletal abnormalities overlying the porencephalic cavities. Fourteen of twenty cases showed evidence of fractures, of which thirteen showed depression of the calvarium and twelve masticatory muscle abnormalities. Of these, seven of fourteen had a history consistent with a head trauma in the first period of life.

**Conclusion:**

The recognition of skull fractures and muscular abnormalities closely associated with the porencephalic cavity may support a diagnosis of a postnatal traumatic origin of porencephaly. Therefore, this study highlights the importance of evaluating musculoskeletal structures in the MRIs of the heads of porencephalic cases.

## 1 Introduction

Cerebral cystic lesions have been described in humans as well as large and small animals. By definition, these brain lesions include extensive lesions such as hydranencephaly, where the neopallium is reduced to a thin, nearly transparent pial-glial-membrane with no associated parenchyma (1), and focal lesions, such as porencephaly. Porencephaly is a less extensive defect, usually represented by a single, cystic, fluid-filled cavity of varying size in the wall of the cerebral hemispheres and typically involves mainly the white matter; connections between the defect and both or either the ventricular and subarachnoid space (2, 3) may be present. In veterinary literature, hydranencephaly and porencephaly are often considered together (4, 5) since parameters for differentiation are not defined, and subjective criteria are sometimes used (6). From an etiopathogenetic point of view, porencephaly is regarded as a congenital disorder and can be classified as a developmental or an encephaloclastic defect. Developmental porencephaly represents a neuronal migration disorder that leaves a defect in the developing cerebral parenchyma. Encephaloclastic porencephaly is a cerebral cavity that results from a destructive process of various etiologies including cerebral ischemia, trauma, and infection (7). Porencephaly is known since decades in veterinary medicine and most of the literature focuses on infectious and metabolic diseases, such as Border disease and copper deficiency, during the fetal life of ruminants and laboratory animals (2, 3, 8–10). In recent years, a few retrospective papers and case reports have been published on canine and feline porencephaly and its diagnosis through magnetic resonance imaging (MRI) (6, 7, 11–14). Certain acquired conditions, including traumatic insults to the brain parenchyma, may also result in focal loss of brain tissue, resulting in cavities that are filled with cerebrospinal fluid (CSF) (“ex vacuo” lesions) and that share the same imaging features as the truly congenital lesions (15). The authors’ hypothesis is that traumatic events in the first weeks or months of life may constitute a cause of canine or feline porencephaly that is more common than generally suspected. The aims of this retrospective study were to (1) investigate a large population of dogs and cats with MRI diagnosis of porencephaly, (2) detect MRI features that might be useful to differentiate presumed postnatal, acquired traumatic from congenital, and (3) define the prevalence of seizure activity.

## 2 Materials and methods

This is a double-center, descriptive, retrospective case series. The medical record databases of the neurology units of two referral veterinary hospitals (AniCura I Portoni Rossi Veterinary Hospital, Zola Predosa, Italy, and Veterinary Teaching Hospital, University of Padua, Legnaro, Italy) were searched for cases between February 2007 and January 2023 of dogs and cats with an MRI-based diagnosis of cerebral cavitary lesions. Terms such as “cysts,” “cavitary lesions,” “ex-vacuo lesions,” “porencephaly,” and “hydranencephaly” were used for the search.

To be eligible for inclusion, dogs and cats needed to have a complete signalment and an MRI of the brain available for reviewing. Data from history and neurological examination, performed by either an ECVN board-certified neurologist or an ECVN neurology resident, were collected when available. In particular, information extracted from the medical records included neuroanatomic localization and neurological signs.

MRIs of the brain were acquired under general anesthesia with either a low-field MRI scanner (0.22 Tesla MrVet, Paramed Medical Systems, Genoa, Italy) or a high-field MRI scanner (1.5 Tesla Vantage Elan, Canon Medical Systems Europe B.V., Netherlands).

All MRI studies were reviewed independently by a European College of Veterinary Diagnostic Imaging (ECVDI) board certified radiologist (C.M.), an American College of Veterinary Radiology (ACVR) board certified radiologist (S.S.), two neurology interns (T.D. and C.L.R.), and a European College of Veterinary Neurology (ECVN) board certified neurologist (M.B.) using a dedicated Digital Imaging and Communications in Medicine viewer program (2020 Horos Project TM). Any discrepancies among observers were resolved by discussion to reach a consensus. Criteria for exclusion from the study were as follows: 1) MRI studies lacking images in all the spatial planes and 2) evidence in the MRI studies of other concomitant brain diseases.

All cases were classified as affected by porencephalic cavities (PCs) when a presumed CSF-filled cystic cavity was located in the wall of a cerebral hemisphere and surrounded at least partially by brain parenchyma (white matter, grey matter, or both). If loss of brain parenchyma for an extension of at least two lobes, associated with a partial loss in other lobes, for an entire hemisphere, or for both hemispheres (6), was identified, the lesion was defined as hydranencephaly and not further considered.

MRI images were assessed for the following: PC number (single, multiple); lateralization (left, right, bilateral); lobar involvement (olfactory, frontal, parietal, piriform, temporal, occipital); communication with CSF spaces (lateral ventricle, subarachnoid space, both, none); involvement of white matter (WM), grey matter (GM), or both; and contrast enhancement (CE – present, absent). When present, concomitant musculoskeletal abnormalities were noted: fractures, thickening or thinning of the overlying calvarium, depression of the calvarium, and ipsilateral masticatory muscle changes (atrophy and fat infiltration). Based on these abnormalities, patients with musculoskeletal abnormalities were classified into two categories: 1) presumed acquired traumatic forms, when fractures and other musculoskeletal changes were visualized, and 2) non-traumatic forms.

When present, further concomitant parenchymal abnormalities were reported. If performed, additional diagnostic tests, such as CSF examination, were registered.

All the findings were entered into a spreadsheet software (Microsoft Excel for Mac, Version 16.74). Descriptive statistics were performed by one of the neurology interns (T. D.).

## 3 Results

Thirty-eight patients were selected after the first search in the medical records. Of these, one case was excluded because it was classified as hydranencephaly, and five cases were excluded because MRIs showed evidence of other concomitant disorders (three suspected necrotizing meningoencephalitis and two neoplasias). Thirty-two cases met the inclusion criteria and were then considered porencephalic cases.

### 3.1 Signalment

Twenty-three cases were dogs, and nine were cats. There were fifteen different dog breeds, with mixed breeds being the most common (8/23). All canine breeds included in the study are listed in Table 1. Thirteen dogs were females (6 neutered), and ten were males (3 neutered). The mean age of the dogs was forty-one months (range 3–142 months), and the median age was twenty-four months. All of the nine cats were domestic shorthairs. Six were neutered females, and three were neutered males. The mean age of the cats was thirty months (range 9–98 months), and the median age was twenty-one months.

**Table 1:** Breeds included in the study.

Dog breeds	Number (%)
Mixed breeds	8 (34.8%)
Border Collie	2 (8.7%)
Bull terrier, Cane Corso, Dobermann, Golden Retriever, Labrador Retriever, Maremma Sheepdog, Pekingese, Pitbull Terrier, German Shepherd, Maltese, Podenco Ibicenco, Chihuahua, French Bulldog	1 (4.3%)

### 3.2 Medical records review

The neuroanatomical localization was available in 28/32 cases. A forebrain localization was made in 28/28 cases, of which it was the only localization in 25/28 cases; in 3/28 cases, it was part of a multifocal localization (peripheral vestibular due to otitis media/interna (n = 2) and T3-L3 spinal cord segments due to spinal cord compression secondary to severe congenital malformations (n = 1)). Sixteen of 28 patients (9 dogs and 7 cats) showed both seizure activity and other neurological deficits. Seven of 28 patients (6 dogs and 1 cat) had a normal neurological examination, and the forebrain localization was hypothesized on the history of seizure activity. Five of 28 cases (4 dogs and 1 cat) were seizure free but showed abnormalities at neurological examination. Table 2 lists all the neurological abnormalities detected in the porencephalic patients. The mean age of the patients at the time of seizure onset was 26.1 months.

**Table 2:** Main clinical signs among the porencephalic patients.

Clinical sign	Number
Seizures	23
Visual deficits	10
Compulsive circling	9
Abnormal behavior	6
Head turn	3
Head tilt	2
Ataxia	2
Horner’s syndrome	1
Inappropriate urination	1
Nystagmus	1
Paraparesis	1

CSF was collected in 11/32 cases and found within normal range (< 5 cell/μL, protein <30 mg/dL) in all cases but one. This was a 9-year-old Pekingese dog showing an albuminocytological dissociation.

### 3.3 MRI features

**Figure 1:**
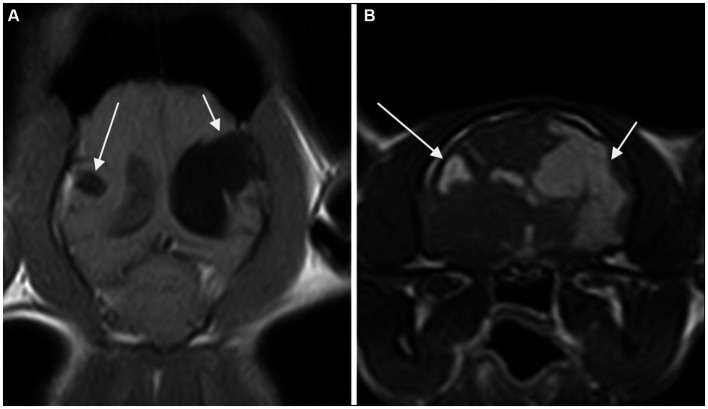
Dorsal T1-weighted **(A)** and transverse 3D-Gradient Echo in Steady State at the level of the thalamus **(B)** MR-images of the brain of a cat revealed two CSF-filled cavities consistent with bilateral porencephaly. A focal lesion located in the right parietal lobe (long arrow) showed communication with neither the ipsilateral lateral ventricle nor the sub-arachnoid space. A second, extensive lesion extending from the left lateral ventricle to the cortical surface at the level of the parietal and temporal lobes (short arrow) is seen.

Twenty-one of 32 patients were examined with a low-field MR scanner and 11/32 with a high-field MR scanner. At least a T2 weighted (T2W) or a T1 weighted (T1W) spin echo (SE) sequence was available in all spatial planes for each MRI study. T1W SE sequences after intravenous administration of 0.2 mL/kg of gadoteric acid were available in all cases. Fluid-Attenuated Inversion Recovery (FLAIR) sequences were available in 27/32 cases. The MRI features of the PCs and musculoskeletal changes are presented in Table 3. A total of thirty-nine PCs were seen. All cases showed either one or two PCs. A single PC was found in 25 (78.1%) cases. In this group, a clear communication with both the ventricular system and the subarachnoid space was seen in 16/25 cases and with the subarachnoid space alone or the ventricular system alone in 7/25 and 2/25 cases, respectively. Two PCs were found in 7 (21.9%) cases (Figure 1), of which both lesions showed a communication with both the ventricular system and the subarachnoid space in four of seven cases. In each of the remaining 3/7 cases, there was a lesion communicating with both the CSF spaces and the other lesion communicating with the subarachnoid space alone (1 case), the ventricular system alone (1 case), or showing no communication at all (1 case). The PCs were confined in a single lobe (6/39) or involved multiple lobes (33/39). The most affected cerebral lobe was the parietal lobe (n = 20), and the least affected was the olfactory lobe (n = 10). The defects involved both the GM and WM in 25/32 cases (78.1%). Contrast enhancement was absent in 27/32 cases and present in 5/32 cases.

**Table 3:** Counts and percentages (in brackets) of the evaluated MRI features within the population study.

MRI features	*n*. (%)
Cases with multiple porencephalic cavities	7/32 (21.9%)
Asymmetrical	7/7 (100%)
Symmetrical	0/7 (0%)
Cases with a single porencephalic cavity	25/32 (78.1%)
Right hemisphere	12/25 (48%)
Left hemisphere	13/25 (52%)
Affected cerebral lobes	
Olfactory lobe	10
Frontal lobe	16
Parietal lobe	20
Temporal lobe	18
Piriform lobe	11
Occipital lobe	17
GM/WM involvement	
Both	25/32 (78.1%)
GM	7/32 (21.9%)
WM	0/32 (0%)
Contrast enhancement	
No	27/32 (84.4%)
Yes	5/32 (15.6%)
Concomitant musculoskeletal abnormalities	
Fractures	14/32 (43.8%)
Thinning	3/32 (9.4%)
Thickening	13/32 (40.6%)
Depression of the calvarium	13/32 (40.6%)
Masticatory muscles changes	13/32 (40.6%)
None	12/32 (37.5%)
Other features	
Meningocele	2/32 (6.2%)
Meningoencephalocele	2/32 (6.2%)
Ipsilateral ventriculomegaly	26/32 (81.2%)
Hydrocephalus internus	10/32 (31.2%)
Absence of septum pellucidum	6/32 (18.7%)
Intracranial arachnoid diverticulum associated with the quadrigeminal cistern/supracollicular fluid accumulation	3/32 (9.4%)

Musculoskeletal changes were observed in 20/32 cases. Fourteen of 20 cases showed evidence of fractures. All these abnormalities were closely related to the PCs.

**Figure 2:**
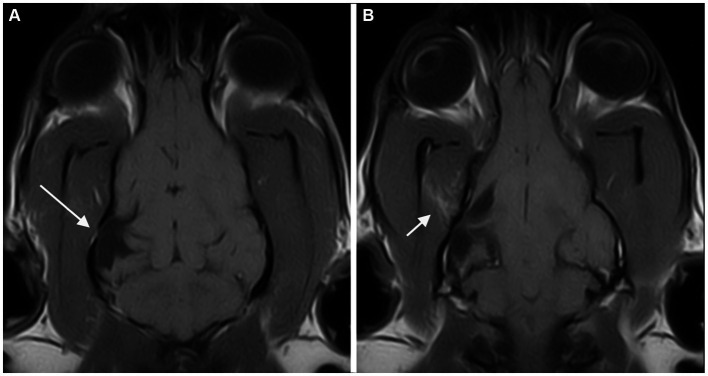
Dorsal T1-weighted MR-images of the brain of a dog showing a porencephalic cavity in the right temporal lobe at level of the caudal collicoli **(A)** and a slice below **(B)**. A temporal bone fracture (long arrow) associated with a mild depression of the overlying calvarium is appreciated. Abnormal signal of the right temporal muscle (fat infiltration) is also present (short arrow).

**Figure 3:**
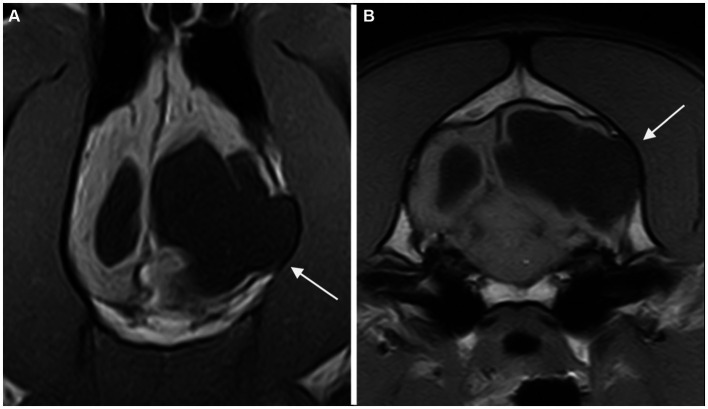
Dorsal **(A)** and transverse at the level of the inner ears **(B)** T1-weighted MR-images of the brain of a dog with an extensive unilateral cavity extending from the left lateral ventricle to the cortical surface. A severe mass effect leading to a right midline shift is present. The cranial vault over the porencephalic cyst is stretched and thinned (arrow).

All fourteen cases with fractures presented at least two further musculoskeletal changes and then were classified as presumed acquired traumatic forms (Figure 2). Thinning of the calvarium overlying the PC was seen in 3 cases (Figure 3). None of them presented further musculoskeletal abnormalities. Depression of the calvarium was seen in 13 cases, all but one of which were associated with fractures. Thirteen cases showed masticatory muscle changes, all but one of which were associated with fractures.

Four of five patients where CE was observed have been classified into the presumed trauma category.

In seven of fourteen cases classified as presumed acquired traumatic forms in MRI, there was a history reporting a head trauma in the first weeks or few months of life: hit by a car (n = 2), bitten/crushed by the mother (n = 1), fell from a height (n = 1), horse kick (n = 1), or clinical evidence of head trauma of unknown origin (n = 2). Two of fourteen cases had no history of trauma, and five of fourteen patients were adopted in adulthood; therefore, history related to the first period of life was not available.

**Figure 4:**
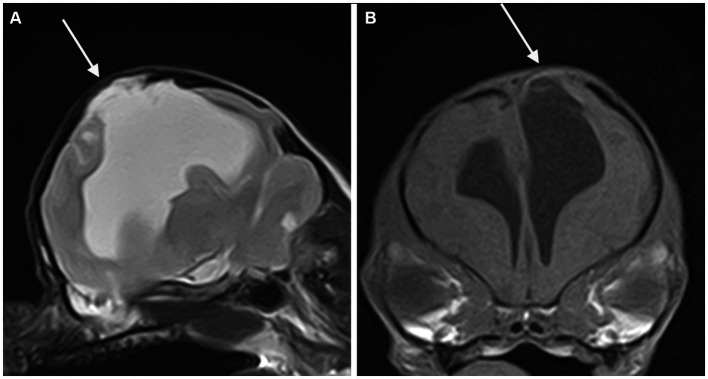
Parasagittal T2-weighted **(A)** and transverse T1-weighted **(B)** MR-images of the brain of a dog showing a porencephalic cavity in the left parietal lobe and associated meningoencephalocele (arrow).

Concurrent abnormalities are listed in Table 3. Meningocele (MC) or meningoencephalocele (MEC) was seen in four cases, all located at the level of the parietal lobe (Figure 4).

## 4 Discussion

This study describes porencephaly in a large population of dogs and cats. Porencephaly is frequently described together with hydranencephaly (5, 6). However, the loss of brain parenchyma in hydranencephaly is severe and differentiates from porencephaly, which is a cavitary lesion with smooth and well-defined borders surrounded by brain tissue (16). Establishing a clear demarcation between these two conditions is challenging, and different definitions have been proposed in both human and veterinary literature in the last decades (4, 17–21). In this study, dogs were considered hydranencephalic when presented with a total parenchymal loss involving at least two lobes and a partial loss in other lobes, as proposed in a recent paper (6).

Porencephaly is poorly described in small animal medicine, especially in cats, with only five cases reported for this species in literature (7, 11, 22, 23). In this study population, 28.1% of the patients were cats, most of them two years old or younger at the time of diagnosis, suggesting that porencephaly should be considered a differential diagnosis in young cats with neurological signs, especially with history of seizure activity.

Moreover, in our population, 21.9% of the patients were 6-year-old or older at the time of diagnosis. Then, independently of the pathogenesis of porencephaly in each of our cases (congenital versus acquired), the related clinical signs could appear late in life (5). Porencephaly may simply represent an incidental finding, as supposed in a previous paper, mainly when the neurological localization of the clinical signs does not match the localization of the PCs (6, 7). However, all twenty-eight patients with an available neuroanatomical localization showed clinical signs of a prosencephalic disorder; in addition, all animals with concurrent neurological disease were previously excluded from this study. This lowers the likelihood that PCs may be an incidental finding.

The main clinical manifestation observed in our population was epileptic seizures (71.8%), as reported in veterinary literature (6, 7, 13, 22). This percentage could even be underestimated since in four cases, information about neurological status was not available. Among the epileptic patients, almost one third had a normal neurological examination, and all but one were aged between twenty-one and seventy-two months. Therefore, our study suggests the importance of considering an MRI of the brain even in patients with an age between six months and six years at seizure onset and a normal neurological examination, for which the first differential diagnosis is idiopathic epilepsy.

Porencephaly is regarded as a congenital anomaly, but it could actually result from a focal injury (vascular accident or trauma) having caused complete loss of a part of the cerebral hemispheres during the perinatal period (16). The World Health Organization defines the perinatal period in human beings as the time frame from twenty-two completed weeks of pregnancy to the first seven days of life (24). A similar definition is lacking in canine and feline medicine. Half of the fourteen patients in our population with MRI abnormalities suggestive of skull fractures had a history of a severe head trauma between the third week and fifth month of life. Concomitant depression of the calvarium was appreciated in all patients but one, and adjacent masticatory muscle lesions were seen in all but two of them, supporting the traumatic etiopathogenesis. These data may be potentially underestimated due to the fact that in this category, the remote history was not available for five of the fourteen cases. On the basis of these findings, if one week of life is accepted as the limit of perinatal conditions in small animals as is the case with humans, then postnatal traumatic events should be considered a possible etiology of this condition.

The association between skull fractures and porencephaly seen in this population could be comparable to the growing skull fractures (GSFs) seen in children as a consequence of head trauma. The pulsatile force of the growing brain causes the skull fracture to enlarge; in addition, interposition of neural tissue prevents healing, inhibiting the migration of osteoblasts to the fracture site. The resorption of the adjacent bone by the continuous pressure from brain tissue herniation through the bone gap adds to the progression of the fracture line; this is the reason why in human medicine, this complication must be corrected early. Porencephaly has been described as a late radiological and surgical finding in patients affected by GSFs (25–27). The same pathogenetic mechanism could explain the relationship between PCs and MC or MEC, as seen in four patients in our study. The congenital lack of the calvarial bone could act as a least resistant point similarly to the skull fracture, allowing the herniation of brain tissue and the developing of the PC. All these malformations occurred at the level of the parietal region; this finding is in accordance with a previous study (28) in which porencephaly was seen in all cases with parietal MC in dogs and with a case report (23) describing a cat with frontoparietal MEC. Another finding that supports a strict relationship between postnatal traumatic brain injury and PCs is that four of the five cases where CE was seen have been classified as presumed acquired traumatic. In these cases, the presence of contrast enhancement could be the result of a chronic blood–brain-barrier dysfunction secondary to traumatic brain injury as reported in both humans and rats in a recent study (29).

The principal limitations of the present study are its retrospective nature and the fact that in almost two thirds of cases, head MRIs were performed with a low field unit. The lack of history for five of the dogs prevents a more exhaustive establishment of a relationship between a possible traumatic event and MRI findings. All the studies were lacking proton density weighted sequences, which are considered the most useful sequences for detecting bone traumatic lesions (30). In addition, the quality of the MRIs obtained with a low field unit could limit the recognition of subtle signs of head trauma, causing one to underestimate the real number of presumed acquired traumatic cases. An additional limitation is the lack of histopathological confirmation in all cases, which might be useful to differentiate congenital from postnatal porencephaly; nonetheless, porencephalic patients are often acceptable pets with a good quality of life, and the diagnosis of porencephaly is generally MRI-based.

In conclusion, this study investigated porencephaly in a large population of dogs and cats. Establishing a possible underlying cause responsible for this condition is often challenging. MRIs can help detect neurological and musculoskeletal abnormalities of the head that support a postnatal, traumatic etiology of the PCs. On the basis of our results, traumatic events are likely to be a more frequent cause of porencephaly than generally reported in literature. Finally, seizure activity is common in porencephalic dogs and cats; porencephaly might be related to seizure activity even when its onset appears in adulthood.

## Data availability statement

The original contributions presented in the study are included in the article/supplementary material, further inquiries can be directed to the corresponding author.

## Ethics statement

Ethical approval was not required for the studies involving animals in accordance with the local legislation and institutional requirements because Ethical review and approval was not required for the animal study because retrospective study. Written informed consent was not obtained from the owners for the participation of their animals in this study because Written informed consent for participation was not obtained from the owners because retrospective study on routine clinical work-up.

## Author contributions

TD: Data curation, Formal analysis, Investigation, Resources, Writing – original draft, Writing – review & editing. CM: Formal analysis, Writing – review & editing. CL: Formal analysis, Resources, Writing – review & editing. CR: Writing – review & editing. SS: Formal analysis, Writing – review & editing. EL: Investigation, Writing – review & editing. ED: Data curation, Investigation, Resources, Writing – review & editing. MB: Conceptualization, Formal Analysis, Project administration, Resources, Supervision, Writing – original draft, Writing – review & editing.
